# Framework for determining airport daily departure and arrival delay thresholds: statistical modelling approach

**DOI:** 10.1186/s40064-016-2623-5

**Published:** 2016-07-08

**Authors:** Ronald Wesonga, Fabian Nabugoomu

**Affiliations:** School of Statistics and Planning, Makerere University, P.O. Box 7062, Kampala, Uganda; Kyambogo University, Kampala, Uganda

**Keywords:** Airport, Delay threshold, Efficiency, Statistical models, Receiver operating curves

## Abstract

The study derives a framework for assessing airport efficiency through evaluating optimal arrival and departure delay thresholds. Assumptions of airport efficiency measurements, though based upon minimum numeric values such as 15 min of turnaround time, cannot be extrapolated to determine proportions of delay-days of an airport. This study explored the concept of delay threshold to determine the proportion of delay-days as an expansion of the theory of delay and our previous work. Data-driven approach using statistical modelling was employed to a limited set of determinants of daily delay at an airport. For the purpose of testing the efficacy of the threshold levels, operational data for Entebbe International Airport were used as a case study. Findings show differences in the proportions of delay at departure (*μ* = 0.499; 95 % CI = 0.023) and arrival (*μ* = 0.363; 95 % CI = 0.022). Multivariate logistic model confirmed an optimal daily departure and arrival delay threshold of 60 % for the airport given the four probable thresholds {50, 60, 70, 80}. The decision for the threshold value was based on the number of significant determinants, the goodness of fit statistics based on the Wald test and the area under the receiver operating curves. These findings propose a modelling framework to generate relevant information for the Air Traffic Management relevant in planning and measurement of airport operational efficiency.

## Background

Airport delay computations are often construed to suit different definitions (Madas and Zografos 2008). Some definitions include aircraft turn-round time, whereas others exclude it. The problem is even larger when one desires to assess daily efficiency of an airport. Many studies have been conducted with the purpose of assessing efficiencies of operations at an airport. In their study of the factors for delays at European airports relative to the airports of the United States of America (Santos and Robin 2014) found that while delays were higher at hub airports, hub airlines experienced lower delays than non-hub airlines. A similar study (Liu et al. 2014) found that there was 30 % greater traffic at airports in the United States of America airports than at European airports that explained more delay at such airports. However, none of the studies considered optimal delay thresholds and its effect on drawing such important conclusions about levels and differences between airports. In his recent study (Wesonga 2015) published the first study that attempted to analyse delay thresholds at airport.

This study introduces the concept of threshold to be employed so as to determine the minimum acceptable proportion above which a day is declared a delay-day at an airport. This study is based on our previous work (Wesonga et al. 2012).

In this paper, data modelling was performed through algorithm design to determine an acceptable threshold for airport delay day (Wong and Tsai 2013; Autey et al. 2013). Furthermore, data modelling was done to a limited set of determinants of delay at an airport for the purpose of testing the efficacy of the threshold levels (Wang et al. 2012; Agustin et al. 2012), using Entebbe International Airport as a case study.

## Data and methodology

Data for the period of 2004 through 2008 were collected on the variables as shown in Table 1. The aviation and aeronautical meteorology variables known to influence airport delay were carefully chosen and tested for autocorrelation before being applied into the modelling process.

**Table 1:** Daily data for aviation and meteorological study parameters for the period 2004 through 2008

Parameter no.	Parameter	Variable type	Daily aggregated data range	
			Minimum value	Maximum value
1	Air temperature	Scale, continuous	19	25
2	Aircraft arriving on time (%)	Scale, discrete	1	42
3	Aircraft delaying arrival (%)	Scale, discrete	0	93
4	Aircraft delaying departure (%)	Scale, discrete	10	89
5	Aircraft on-time departure (%)	Scale, discrete	0	81
6	Chartered flights	Scale, discrete	0	50
7	Dew point temperature	Scale, continuous	16	21
8	Freighters	Scale, discrete	0	12
9	Non-commercial flights	Scale, discrete	0	57
10	Persons on board-in	Scale, discrete	138	3128
11	Persons on board-out	Scale, discrete	130	3277
12	Queen’s nautical height	Scale, continuous	975	1098
13	Scheduled flights	Scale, discrete	5	55
14	Visibility	Scale, continuous	7558	9999
15	Wind direction	Scale, discrete	107	329
16	Wind speed	Scale, discrete	2	9

For each day at an airport, there are registered levels of delay. These vary in proportions over time and would be misleading if one performed analysis based on the consideration that any positively registered delay at an airport is actually a delay in its real sense. Some delays are meant to enable an aircraft perform more efficiently throughout its trajectory with minimum disturbances and distortions such as being re-routed through other airports or even being cancelled. Therefore, if not all delays are bad in the real sense, a question of what proportion of delay should be treated as a threshold for computational and modelling purpose became eminent and a subject for this study.

## Statistical model framework

Modelling was premised on the fact that different levels of thresholds could dynamically affect the statistical significance of determinants for airport delay. The question of their levels of influence was studied using generalised linear models as demonstrated in Eqs. (1), (2) and (3).

Logistic regression model with dummies ‘0’ for airport’s daily on-time performance while and ‘1’ for daily airport delay, constituted the dependent variable (Konishi and Kitagawa 2007; Nerlove and Press 1973). Determining what threshold to apply in this generalised linear modelling was an area of interest for this study. An aircraft is said to have delayed if the difference between the actual and scheduled times of arrival or departure were positive. In this study, a value for the dependent variable change based on what threshold is applied. The threshold start point was a proportion of 1 % and the ultimate being 100 % which implied that on any given day for any reporting based on the chosen proportion (1 through 100 %) of delay, such a day would be classified as a delay-day (DD) otherwise not-delay-day (NDD). Note that the daily proportions of delay were obtained by dividing the number of aircrafts that delay their operation by the total number for such an operation multiplied by one hundred; the operations could be departures or arrivals.

Furthermore, a logistic regression model, known to estimate the probability with which a certain event would happen or the probability of a sample unit with certain characteristics expressed by the categories of the predictor variables, to have the property expressed by the value 1 representing an airport’s delay day was employed. Estimation of the probability was done by the logistic distribution as in Eq. (2), where *β*’s are the regression coefficients of the categories to which the sample unit belongs.

The following formulation was deemed as appropriate for modelling departure and arrival delay.

1 $$\ln \left( {\frac{{\uppi\left( {{\text{X}}_{i} } \right)}}{{1 -\uppi\left( {{\text{X}}_{i} } \right)}}} \right) = \mathop \sum \limits_{{{\text{j}} = 1}}^{\text{p}}\upbeta_{\text{j}} {\text{X}}_{\text{ij}}$$

where β_j_ represent coefficients of the model; ${\text{X}}_{\text{i}} = \left\{ {{\text{X}}_{{{\text{i}}1}} ,{\text{X}}_{{{\text{i}}2}} , \ldots ,{\text{X}}_{\text{ip}} } \right\}$ represent a set of explanatory variables.

The logit ${ \ln }\left( {\frac{{\uppi\left( {{\text{X}}_{i} } \right)}}{{1 -\uppi\left( {{\text{X}}_{i} } \right)}}} \right)$ on the left hand side of Eq. (1) represent the logarithm of the odds ratio which symbolize the conditional probability for DD given a set of explanatory variables and its determinants were subsequently tested for significance of the underlying relationship.

2 $$\frac{{\uppi\left( {{\text{X}}_{i} } \right)}}{{1 -\uppi\left( {{\text{X}}_{i} } \right)}} = { \exp }^{{ \mathop \sum \nolimits_{{{\text{j}} = 1}}^{\text{p}}\upbeta_{\text{j}} {\text{X}}_{\text{ij}} }}$$

Therefore, the odds are exponential function of ${\text{X}}_{\text{i}}$ that provided a basic interpretation of the magnitude of the coefficients. Positive $\upbeta_{\text{j}}$’s imply an increasing rate while negative $\upbeta_{\text{j}}$ implies a decreasing rate and in either way, the magnitude of $\upbeta_{\text{j}}$ show the effect or level of contribution towards determining DD. On the contrary, if $\upbeta_{\text{j}} = 0$ then the airport’s DD was said to be independent of ${\text{X}}_{\text{i}}$.

3 $$\uppi\left( {{\text{X}}_{\text{i}} } \right) = \frac{{{ \exp }^{{ \mathop \sum \nolimits_{{{\text{j}} = 1}}^{\text{p}}\upbeta_{\text{j}} {\text{X}}_{\text{ij}} }} }}{{1 + { \exp }^{{ \mathop \sum \nolimits_{{{\text{j}} = 1}}^{\text{p}}\upbeta_{\text{j}} {\text{X}}_{\text{ij}} }} }}$$

Note that the values $0 \le \pi \left( {{\text{X}}_{\text{i}} } \right) \le 1$ represent the probability of delay-day based on a set of meteorological and aviation parameters as shown in Table 1.

Since the logistic regression model is known to exhibit a curve rather than a linear appearance, the logistic function implied that the rate of change in the odds $\uppi ( {\text{X}}_{\text{i}} )$ per unit change in the explanatory variables ${\text{X}}_{\text{i}}$ varied according to the relation $\frac{{\partial {\pi (}{\text{X}}_{\text{i}} \text{)}}}{{\partial \text{(}{\text{X}}_{\text{i}} \text{)}}} =\upbeta_{j} [{\pi (}{\text{X}}_{\text{i}} \text{)}]\,[1 - {\pi (}{\text{X}}_{\text{i}} \text{)}]$. For example, if the odds of the proportion of delay $\uppi\left( {{\text{X}}_{\text{i}} } \right) = \frac{1}{2}$ and the coefficient of the number of ‘scheduled flights’ $\upbeta = 0.46$, then the slope $\frac{{\partial {\pi (}{\text{X}}_{\text{i}} \text{)}}}{{\partial \text{(}{\text{X}}_{\text{i}} \text{)}}} = 0.46 * \frac{1}{2} * \frac{1}{2} = 0.115$. The value 0.115 represents a change in the odds of departure delay, $\uppi ( {\text{X}}_{\text{i}} )$ per unit change in the number of ‘scheduled flights’. In simpler terms, for every 100 scheduled flights at Entebbe International Airport, 11 delay to departure. The R platform for statistical computing scientists (Chambers 2008; Dalgaard 2008) was applied because of its known strengths in computing that include, but not restricted to: the most comprehensive statistical analysis package available because it incorporates all of the standard statistical tests, models and analyses, as well as provides a comprehensive language for managing and manipulating data.

## Findings and discussions

### Data structure

Over the period under study, on every day, the total number of aircrafts departing and arriving at Entebbe International airport was recorded. For each departure and arrival, each aircraft’s operational performance was assessed in terms of the scheduled and actual times and thus categorised accordingly. Thus, on every day and for every N aircrafts at the airport, there were $N_{D}$ and $N_{A}$ departures and arrivals respectively. And for every $N_{D}$ and $N_{A}$, some $N_{Dd}$ or $N_{Ad}$ and $N_{Dt}$ or $N_{At}$ were computed to represent either departure or arrival delays and on-time departure or arrival respectively. Therefore, on an ith day, the following computations were derived where the proportions for daily aircraft departures and arrivals were computed on the one to one relationship;

4 $$\left( {\begin{array}{*{20}c} {P_{Dd} } \\ {\begin{array}{*{20}c} {P_{Dt} } \\ {P_{Ad} } \\ {P_{At} } \\ \end{array} } \\ \end{array} } \right) = \left( {\begin{array}{*{20}c} {\frac{{N_{Dd} }}{{N_{Dd} + N_{Dt} }}} \\ {\frac{{N_{Dt} }}{{N_{Dd} + N_{Dt} }}} \\ {\frac{{N_{Ad} }}{{N_{Ad} + N_{At} }}} \\ {\frac{{N_{At} }}{{N_{Ad} + N_{At} }}} \\ \end{array} } \right) \times 100\,\%$$

Subsequently, for any ith day, a decision was taken to categorise it as a delay-day, DD or not a delay day, NDD based on a set of delay thresholds *dT* = {10, 20, 30, 40, 50, 60, 70, 80, 90, 100}. However, the decision to determine a DD for departures and arrivals was based on the following one to many comparisons below;

5 $$\left( {\begin{array}{*{20}c} {P_{Dd} } \\ {P_{Ad} } \\ \end{array} } \right) \ge \left( {\begin{array}{*{20}c} {50} \\ {60} \\ {70} \\ {80} \\ \end{array} } \right)$$

The delay thresholds *dT* = {10, 20, 30, 40, 90, 100} were found inappropriate to model because they are logically not suitable since values for delay proportions less than 50 % could imply that on time performance was more than delay and 90 with 100 % tended to imply that all flights delayed, which in our case study did not arise on any day.

### Descriptive statistics for the dependent dummy threshold levels

To be able to employ the logistic regression modelling approach, we thus created dummy variables for departure and arrival for each of the four candidate delay thresholds *dT* = {50, 60, 70, 80} as $dT = \left\{ {dT50, dT60, dT70, dT80} \right\}$ and $aT = \left\{ {aT50, aT60, aT70, aT80} \right\}$ respectively. Table 2 shows the descriptive statistics for the candidate departure and arrival delay thresholds.

**Table 2:** Descriptive statistics for candidate threshold dummy variables

	Departure delay thresholds				Arrival delay thresholds			
	*dT50*	*dT60*	*dT70*	*dT80*	*aT50*	*aT60*	*aT70*	*aT80*
Mean	0.945	0.499	0.267	0.051	0.829	0.363	0.182	0.044
Standard error	0.005	0.012	0.010	0.005	0.009	0.011	0.009	0.005
Standard deviation	0.229	0.500	0.442	0.221	0.377	0.481	0.386	0.206
Sample variance	0.052	0.250	0.196	0.049	0.142	0.231	0.149	0.042
Kurtosis	13.187	−2.002	−0.884	14.533	1.050	−1.679	0.715	17.654
Skewness	−3.895	0.005	1.057	4.064	−1.746	0.568	1.647	4.431
Sum	1726	911	487	94	1514	664	333	81
Count	1827	1827	1827	1827	1827	1827	1827	1827
Confidence level (95.0 %)	0.010	0.023	0.020	0.010	0.017	0.022	0.018	0.009

From Table 2, examining the candidate thresholds for departure delay descriptive statistics, for one to get an unbiased threshold, it was desirable that the statistics point at the middle values as much as possible. In the event that there was no one candidate presenting the desired exact middle values, then the threshold candidate with values approximating the middle characteristics was preferred. Therefore, preliminary findings in this study based on the actual operational data at Entebbe International Airport both for departure ($\bar{X} = 0.499; \,SE = 0.012)$ and arrival ($\bar{X} = 0.363; \,SE = 0.011)$ delay thresholds propose for recommendation a delay thresholds of 60 % (Ivanov et al. 2012).

### Algorithm for determination of thresholds for departure and arrival delays

In Table 3, a set of processes for the algorithm employed to take care of the computational procedure of the study is presented.

**Table 3:**
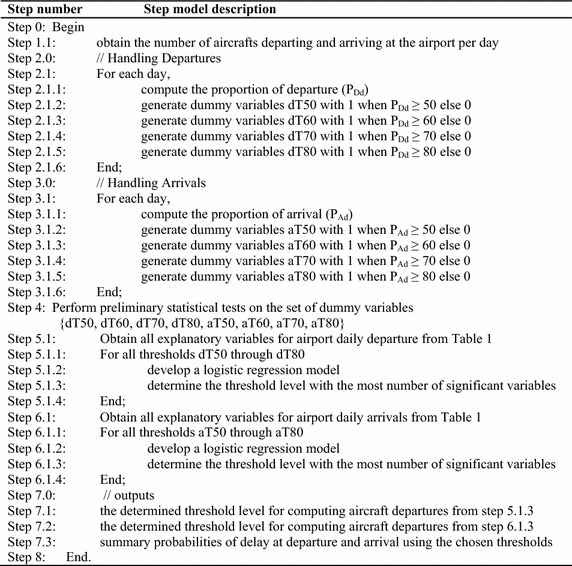
General algorithm for determining suitable thresholds for departure and arrival delays

### Departure delay determinants

Table 4 presents the adjusted odds ratios for the logistic models under different prior thresholds showing the levels of significance for the determinants of departure delay. All the four threshold values were assumed with the Wald goodness of fit test-statistics computed for each model representing a certain threshold level. The areas under the ROC curves were presented.

**Table 4:** Model estimations based on four threshold levels for departure delay determinants

Characteristic	Adjusted odds ratio			
	Threshold 1 (50 %)	Threshold 2 (60 %)	Threshold 3 (70 %)	Threshold 4 (80 %)
Arrival threshold	2.871**	0.457**	0.203**	1.000
Arrival delay	0.891**	1.011	1.003	0.978
Aircraft operations	0.541*	1.288**	1.810**	4.815
Scheduled flights	1.910*	0.651**	0.466**	0.002
Chartered flights	1.723*	0.635**	0.485**	0.002
Freighters	2.145**	0.827**	0.598**	0.002
Non-commercial	2.224**	0.842**	0.584**	0.002*
Persons outgoing	0.999*	1.000	1.001**	1.002
Persons incoming	1.001	1.000	1.000	0.999
Visibility	0.999	0.999*	1.000	1.000
Wind speed	1.003	1.038	1.039	1.005
Constant	4.794	62.914**	1.710	0.339
Observations (N)	1827	1827	1827	1827
Covariate patterns	1827	1827	1827	1827
Pearson chi^2^	3312.400	1703.820	2000.380	1092.990
Prob > chi^2^	0.000	0.970	0.001	1.000
Area under ROC curve	0.841	0.887	0.875	0.807

* represents 0.05 and ** represents 0.01 statistical levels of significance

**Fig. 1:**
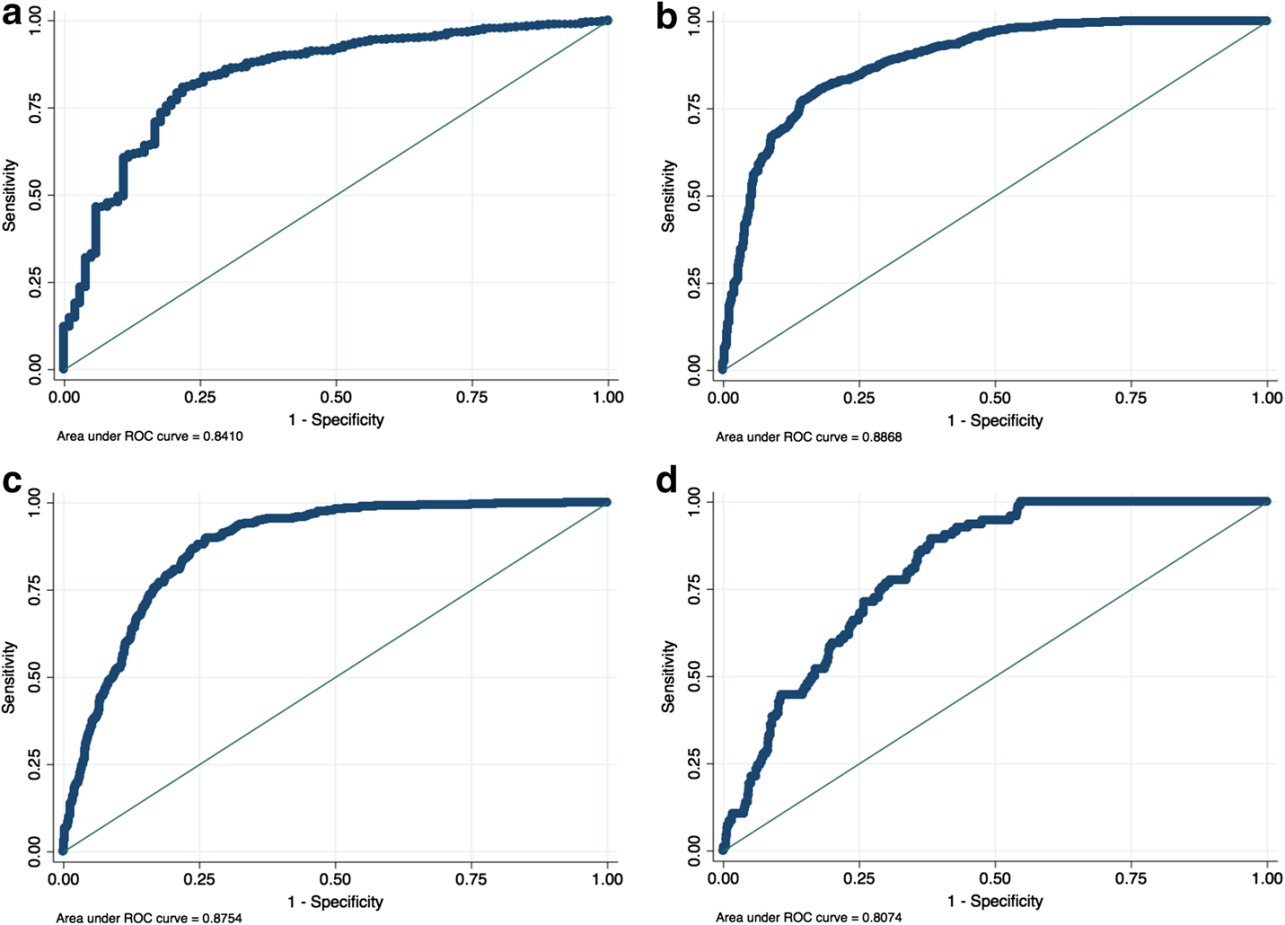
Area under ROC curve for different departure delay thresholds. **a** 50 % departure threshold, **b** 60 % departure threshold, **c** 70 % departure threshold, **d** 80 % departure threshold

The effects of parameters on departure delays was examined as shown in Table 4. Model coefficients were examined for all determinants of departure delay that were generated at the four candidate threshold values (50, 60, 70, 80). The Wald test-statistics were examined for each model for statistical significance at the four candidate threshold levels. The criterion for selection of the best model and thus the most appropriate threshold level was done based on the variable qualities; besides the Wald test-statistics and the area under the ROC curve as shown in Fig. 1. As a result, the delay threshold of 60 % was found to generate the best model, followed by 70, 50 and 80 % respectively.

**Fig. 2:**
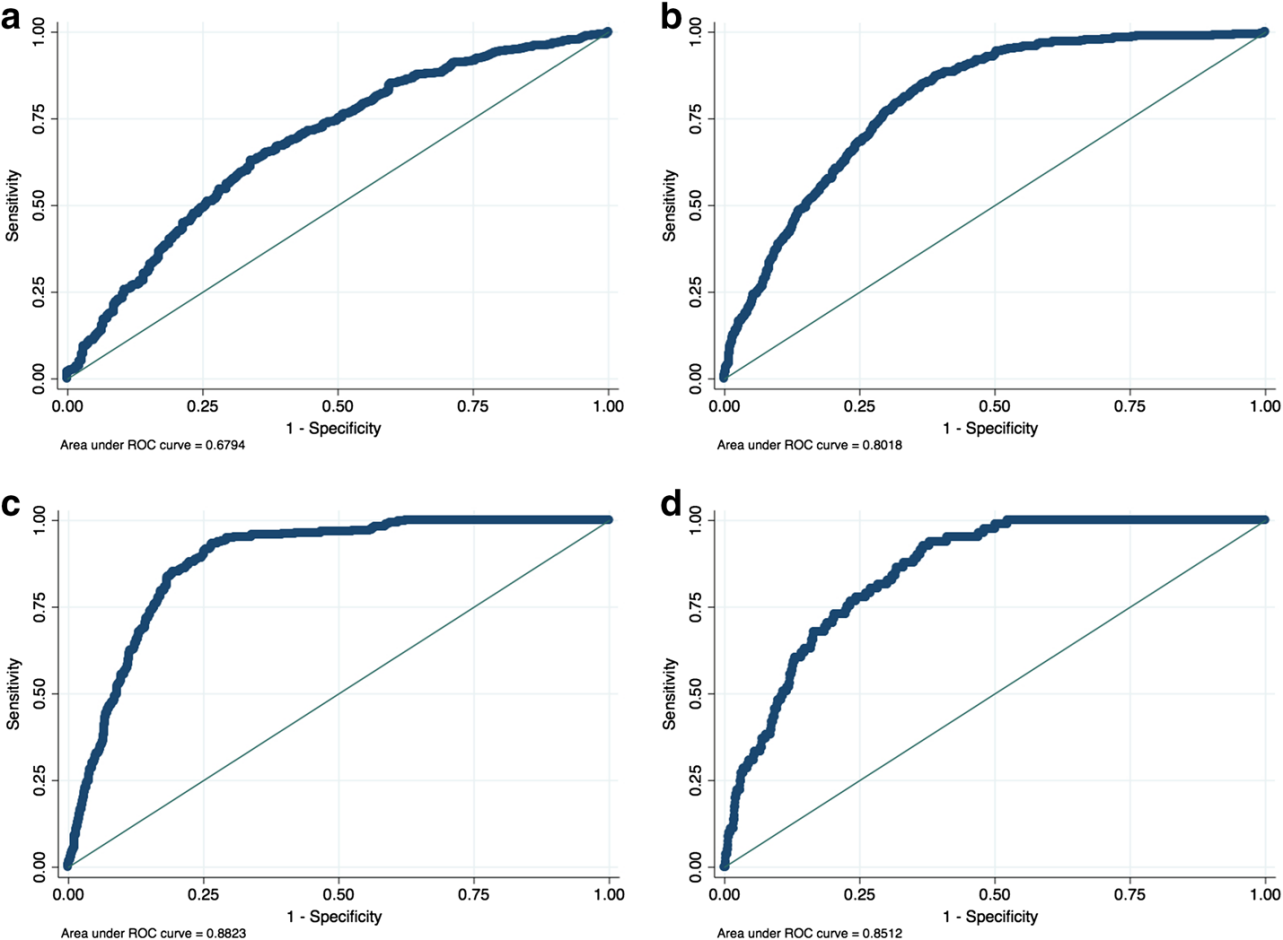
Area under ROC curve for different arrival delay thresholds. **a** 50 % arrival delay threshold, **b** 60 % arrival delay threshold, **c** 70 % arrival delay threshold, **d** 80 % arrival delay threshold

Table 5 presents models at the different levels of significance for determinants of arrival delay. All the four threshold values (50, 60, 70, 80) were assumed and estimates of the logit model computed at every level. The Wald test-statistics were examined for each model and statistical significance for the predictors at the four candidate threshold levels. The quality of variables; the Wald test-statistics and the area under the ROC curves as shown in Fig. 2 were applied to determine the best model. As a result, the delay threshold of 60 % was found to generate the best model, followed by 70, 50 and 80 % respectively.

**Table 5:** Arrival delay determinants model based on four threshold levels

Characteristic	Adjusted odds ratio			
	Threshold 1 (50 %)	Threshold 2 (60 %)	Threshold 3 (70 %)	Threshold 4 (80 %)
Departure threshold	0.578	0.248**	0.137**	1.000
Departure delay	1.012	1.087**	1.028	0.936*
Number of operations	0.965	1.462**	1.960**	2.984
Scheduled flights	1.035	0.577**	0.000	0.000
Chartered flights	1.052	0.650**	0.001**	0.000
Freighters	0.883*	0.534**	0.001**	0.000
Non-commercial	1.057	0.660**	0.001**	0.000
Persons outgoing	1.000	1.001	1.001*	1.001
Persons incoming	1.001*	1.000*	1.001**	1.001
Visibility	1.000	0.999*	1.000	
Wind speed	0.976	1.025		
Constant	1.097	21.430**	4.618	0.880
No. of observations	1827	1827	1827	1827
No. of covariate patterns	1827	1827	1827	1827
Pearson chi^2^	1874.690	2648.530	1352.250	1119.900
Prob > chi^2^	0.161	0.000	1.000	1.000
Area under ROC curve	0.679	0.882	0.802	0.844

* represents 0.05 and ** represents 0.01 statistical levels of significance

## Discussions and conclusions

We explored modelling approach premised on the binary logistic regression to determine a better level of delay threshold that optimally evaluates the dynamics of air traffic delay during departure and arrival at an airport (Santos and Robin 2010). Four different scenarios were evaluated for both cases of departures and arrivals. The study established that at Entebbe International Airport, departure delay threshold of air traffic flow operations of 60 % provided the best and stable model characteristics. Variations of levels of significance for parameters of delay were detected at different delay thresholds, thus generating different numbers of significant parameters. For example, in both Tables 4 and 5; sub-table (d) presented the worst levels of parameter sensitivity with the least number of significant variables while sub-table (b) provided more stable models in both cases (Wesonga and Nabugoomu 2014; Helmuth et al. 2011).

These findings are significance in two ways; first, to the air traffic flow managers that daily proportions of aircraft delay below the 60 % threshold level could be considered normal operations. Therefore, such daily delays may be attributed to normal airport operational such as the turn-around time before actual departures and arrivals. Secondly, to the other aviation stakeholders including air passengers, the higher threshold level would indicate inefficiency of traffic flows. Comparison of air traffic flow inefficiencies based on the findings for departures are in the threshold order of 60 %, then 70 % compared to arrival threshold of 60 % followed by 50 % indicating that traffic flow at arrival was less inefficient than that during departure since arrivals permitted lower threshold level than departures (Wesonga et al. 2013; Zheng et al. 2010).

Besides, comparing aircraft flow performance between daily departures and arrivals, this framework is candidate to providing methodology for assessment and ranking of airports based on their departure and arrival operational efficiency. Airports with derived higher delay thresholds would be assessed as operationally more inefficient than those with lower delay thresholds (Chou 2009; Wei et al. 2011). Therefore, a multi-airport analysis based on this framework is recommended as a possible area of further analysis and application of the derived framework of this study (Mukherjee and Hansen 2009; Bianco et al. 2001).
